# Successful Diagnosis of Hypothalamitis Using Stereotactic Biopsy and Treatment: A Case Report

**DOI:** 10.1097/MD.0000000000000447

**Published:** 2015-02-06

**Authors:** Shuo Zhang, Hongying Ye, Zhaoyun Zhang, Bin Lu, Yehong Yang, Min He, Hanfeng Wu, Linuo Zhou, Yin Wang, Li Pan, Yiming Li, Renming Hu

**Affiliations:** From the Department of Endocrinology and Metabolism (SZ, HY, ZZ, BL, YY, MH, LZ, YL, RH); Department of Neuropathology (YW), Huashan Hospital; and Department of Neurosurgery (HW, LP), Huashan Hospital and Shanghai Gamma Hospital, Fudan University, Shanghai, China.

## Abstract

Existing methods could not discriminate between inflammation and other diseases, which might occur in hypothalamus, such as neurogliocytoma, germinoma, lymphoma, and so on. Given its location in the brain, it was not practical to obtain tissue using standard surgical methods.

We reported the first case of a patient with hypothalamus lesion, who was diagnosed as hypothalamitis by stereotactic biopsy. This precise diagnosis allowed proper medical treatments.

We reported a case of a patient with hypothalamus lesion. To confirm the diagnosis, with informed consent from the family, a successful stereotactic hypothalamic biopsy was performed by neurosurgeons.

Immunohistochemical results of biopsy specimens from the hypothalamus lesion revealed inflammatory infiltrates, which were composed mainly of lymphocytes, plasma cells, and histiocytes, and were stained with leucocyte common antigen (LCA), κ 1, and cluster of differentiation 18. Final pathological diagnosis was lymphoplasmacytic proliferative, granuloma-like inflammatory pseudotumor, with immunoglobulin G deposition. Based on the pathological diagnosis, we treated the patient with glucocorticoid and azathioprine. Remarkable improvements were observed in both magnetic resonance imaging (MRI) and patient's symptoms.

Stereotactic biopsy for intracranial lesions was a reliable and relatively safe procedure, even for hypothalamus. It was an effective method with high diagnostic yield. With correct diagnosis, it was much easier to choose correct treatment.

## INTRODUCTION

Diseases of hypothalamus could cause pituitary dysfunction, neuropsychiatric and behavioral disorders, and disturbances of autonomic and metabolic regulation. The etiology of hypothalamic neuroendocrine disorders (for 10–25 years) included tumors (craniopharyngioma, dysgerminoma, glioma, dermoid, leukemia, and neuroblastoma), trauma, inflammatory disease (meningitis, encephalitis, sarcoidosis, tuberculosis, etc), and vascular (subarachnoid hemorrhage, aneurysm, and arteriovenous malformation) and structural brain defects. Treatment varied with the underlying disease, therefore, it was very important to obtain a correct diagnosis. Existing methods could not discriminate between inflammation and other diseases, which might occur in hypothalamus, such as neurogliocytoma, germinoma, lymphoma, and so on. Given its location in brain, it was hard and dangerous to obtain tissue using standard surgical methods. Here we reported the first case of hypothalamic syndrome caused by hypothalamic inflammatory granuloma, diagnosed by stereotactic hypothalamic biopsy.

### Case Report

**FIGURE 1:**
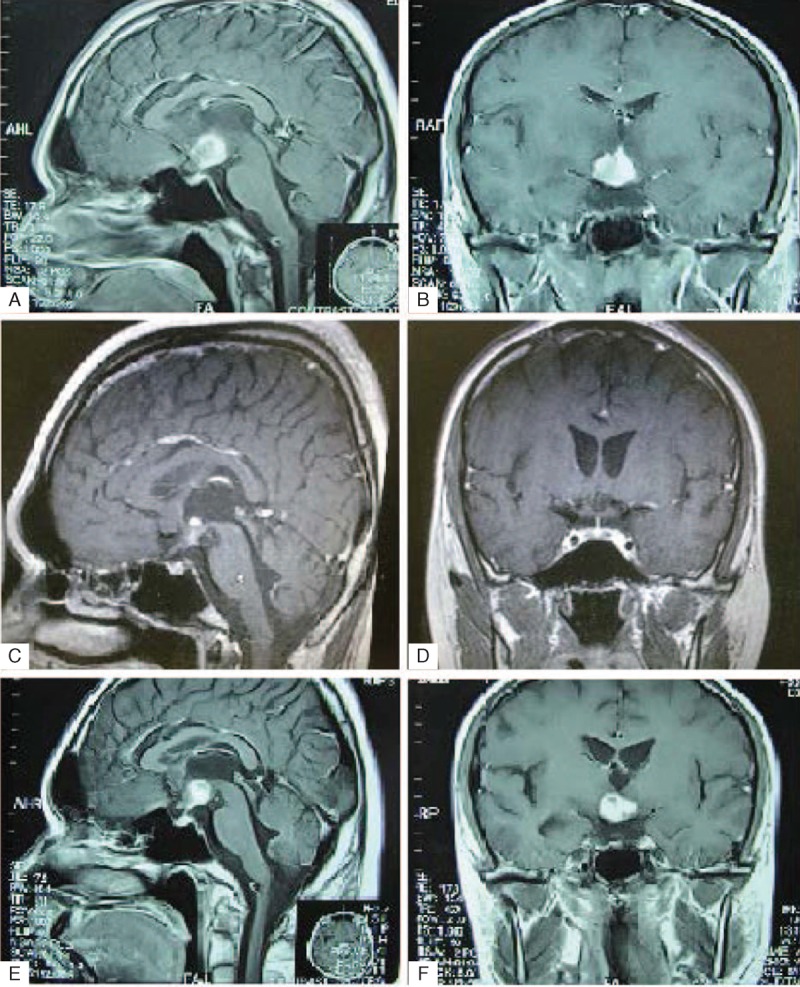
MRI Scans of the patient in Suzhou hospital. Panels A and B show initial imaging, before any treatments. MRI revealed a space-occupying lesion in the hypothalamus. MRI showed decrease in size of the lesion after steroid treatment (Panels C and D). When dexamethasone was gradually withdrawn (to 2.25 mg), the patient's symptoms worsened, and the lesion was seen to enlarge on MRI (Panels E and F). MRI = magnetic resonance imaging.

A 20-year-old male college student had sudden onset of polyphagia, polyuria, polydipsia, deterioration of memory, and personality change, 2 months before hospital admission in July 2010. His urine volume was 6000 to 8000 mL every day, and he preferred cool water. He had poor memory and easy fatigability. He was admitted into Suzhou hospital where magnetic resonance imaging (MRI) revealed a hypothalamus lesion (Figure 1A and B). Inflammation, neurogliocytoma, or germinoma was considered in the differential diagnoses. Cerebrospinal fluid was normal, but serum cortisol was only 1.23 μg/dL (normal range 6.20–19.40 μg/dL). The patient was treated with intravenous administration of desmopressin and antibiotics. He was also administered dexamethasone by intravenous drip, followed by oral administration. The patient's symptoms improved and MRI demonstrated decrease in size of the lesion (Figure 1C and D). When oral administration dosage of dexamethasone was gradually decreased (to 2.25 mg once a day), the patient became more symptomatic and MRI demonstrated an increase in the size of the lesion (Figure 1E and F). Oral administration of prednisone 20 mg once a day was then started. Several days later, the patient developed an evening fever, which could not be relieved by antipyretics and antibiotics. The patient was transferred to the Department of Endocrinology of Huashan Hospital. On admission, the patient's symptoms were unexplained fever, polyphagia, polyuria, polydipsia, deterioration of memory, and personality changes. His consciousness was clear but lethargic. He did not have any medical history. Physical examination was normal except for “purple striae” on both sides of his waist. Endocrine evaluation showed severe disturbances (Tables 1 and 2). A dehydration test could not be completed because the patient was uncooperative. Primary diagnoses were hypothalamic syndrome of unknown cause with panhypopituitarism, diabetes, and dyslipidemia. The patient was treated with oral intake of 25 mg cortisone once a day, 50 μg levothyroxine sodium once a day, 400 μg desmopressin (100 μg–150 μg–150 μg), and metformin. MRI at that time demonstrated a lesion in the hypothalamus.

**TABLE 1:**
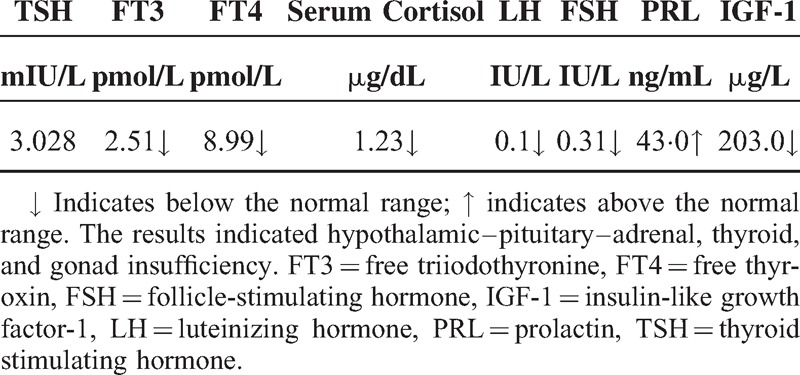
Endocrine Findings

**TABLE 2:**
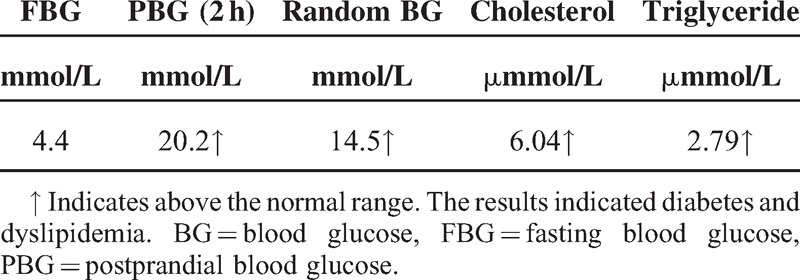
Endocrine Findings

## METHODS

Because of the broad differential diagnosis, with informed consent from the family, we decided to perform a stereotactic hypothalamic biopsy (Dr P.L. and W.H.F.) to confirm the diagnosis.

Under general anesthesia, the patient's head was fixed using a Leksell G head frame. MRI was used to determine the target point and to calculate the 3-dimensional location of the lesion. An incision was performed at the right forehead. The biopsy needle was advanced to a point 10 mm from the center of the lesion, and biopsies were taken. Biopsy specimens were obtained every 3 mm as the needle was advanced, until the needle was 3 mm deep from the center of the target. The biopsy needle was rotated to obtain specimens from 8 different quadrants along the long axis of the needle. There was no active bleeding after the procedure.

## RESULTS

**FIGURE 2:**
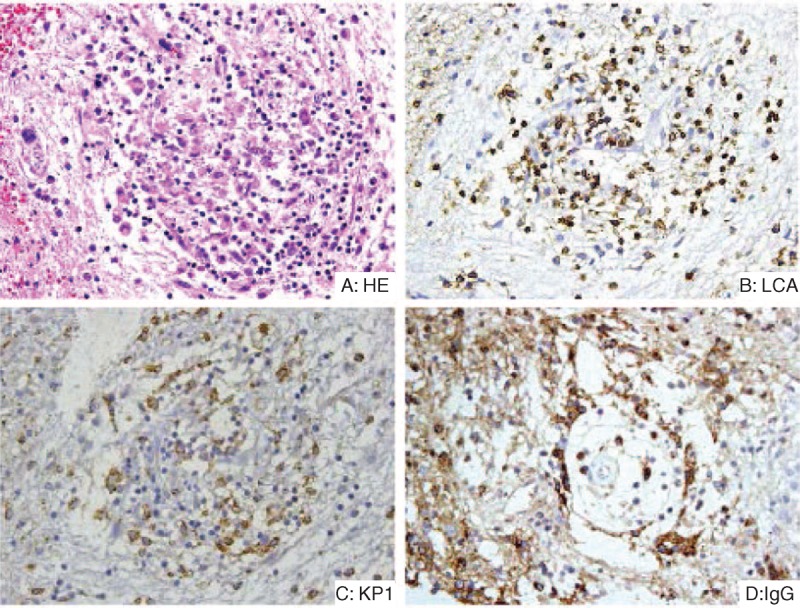
Biopsy specimens of the hypothalamus. Aggregation and infiltration of inflammatory cells, mainly lymphocytes and plasma cells, and some histiocytes, were seen in the biopsy specimens. Panel A shows hematoxylin-eosin staining (×400); Panel B shows scattered LCA-positive lymphocytes (×400); Panel C shows scattered KP1-positive plasma cells (×400); Panel D shows significant IgG-positive cell infiltration (×400). IgG = immunoglobulin G, KP1 = κ 1, LCA = leucocyte common antigen.

Microscopic examination of the biopsy specimens revealed inflammatory infiltrates, which were composed mainly of lymphocytes, plasma cells, and histiocytes (the biopsy specimens were stained with leucocyte common antigen, κ 1, and cluster of differentiation 18). Final pathological diagnosis was lymphoplasmacytic proliferative granuloma-like inflammatory pseudotumor with immunoglobulin G (IgG) deposition (Figure 2). A new disease entity consisting of hypophysitis associated with IgG4-related systemic disease had been described.^1–4^ This was part of our differential diagnosis, although IgG4 stain result of the biopsy specimens was negative.

The patient was treated with 5 mg dexamethasone by intravenous administration once a day for 20 days after surgery. After that, dexamethasone was changed to oral prednisolone 16 mg (8:00 hours) and 12 mg (16:00 hours), and then prednisolone was tapered 8 mg every week. At the same time, oral azathioprine^5^ was added as 25 mg twice a day. A week later, this was increased to 50 mg twice a day for maintenance. When oral prednisolone was tapered to 4 mg (8:00 hours) and 2 mg (16:00 hours), oral hydrocortisone 20 mg (8:00 hours) and 10 mg (16:00 hours) were substituted for replacement therapy.

**FIGURE 3:**
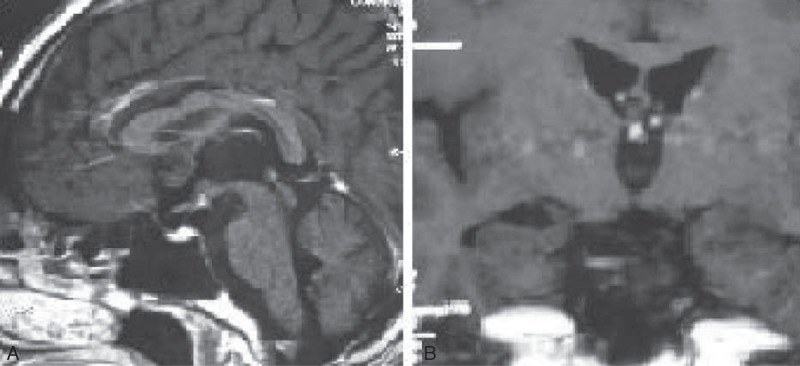
Latest MRI of the patient (almost 2 years after the treatment was given). MRI showed that the abnormality in the hypothalamus on MRI has markedly improved. MRI = magnetic resonance imaging.

One year later, azathioprine was tapered to 50 mg once a day by oral administration for 6 months, and then it was stopped until now. The latest MRI showed that the hypothalamic lesion was markedly decreased in size (Figure 3). Current medications are 50 μg levothyroxine sodium once a day, hydrocortisone 10 mg (8:00 hours) once a day, and desmopressin 50 μg once a day by oral administration. At the latest follow-up, the patient's blood glucose was normal without any treatment.

The patient now lives a normal life and has returned to school. Till now (for almost 4 years), we have not found any adverse or unanticipated events of the patient. The patient and his parents show their satisfaction with the treatments.

## DISCUSSION

Hypothalamus is one of the most essential regions of the brain. It secretes many hormones essential for life, even though it is very small, <1% of the whole brain weight. Any hypothalamic lesion could cause hypothalamic syndrome. Given its location in the brain, it was hard and dangerous to obtain tissue using standard surgical methods. Although patients with clinical hypophysitis had been reported^6–11^), there was no biopsy confirmation report because of difficulty in obtaining and confirming the pathology of the lesion.^12,13^ Here, we performed a stereotactic hypothalamic biopsy to confirm the diagnosis.

Stereotactic biopsy for intracranial lesions was a reliable and relatively safe procedure. It was also a very efficacious method, with a high diagnostic yield, especially in patients who needed histological confirmation for treatment.^14,15^ The selection criteria for stereotactic biopsy comprise the patients whose lesions were located in obvious or deep-seated areas. Usually, we excluded the cases with severe neurological deformities whose radiological findings showed increased intracranial pressure and the patients with bleeding tendency that could not be controlled. However, the risks of stereotactic biopsy for hypothalamus were postoperative intratumoral hematoma and increased edema without hemorrhage. Therefore, stereotactic hypothalamic biopsy should be performed by experienced neurosurgeon, and special care should be taken to prevent hemorrhage after biopsy. The outcome of this biopsy also depended on the lesion of hypothalamus, such as size, shape, and so on.

Based on the histologic results, the lymphoplasmacytic proliferative, granuloma-like inflammatory pseudotumor, with IgG immunoglobulin deposition was diagnosed. After the successful treatments based on correct diagnose, a remarkable mass reduction was observed by MRI, and the patient's symptoms were markedly improved.

This was the first case of an inflammatory granuloma of hypothalamus confirmed by stereotactic hypothalamic biopsy. This diagnosis was supported by biopsy findings, which guided successful treatment.

## References

[1] Tanabe T Tsushima K Yasuo M IgG4-associated multifocal systemic fibrosis complicating sclerosing sialadenitis, hypophysitis, and retroperitoneal fibrosis, but lacking pancreatic involvement. *Intern Med* 2006; 45:1243–1247. 17139126 10.2169/internalmedicine.45.1759

[2] Yamamoto M Takahashi H Ohara M A case of Mikulicz's disease (IgG4-related plasmacytic disease) complicated by autoimmune hypophysitis. *Scand J Rheumatol* 2006; 35:410–411. 17062446 10.1080/03009740600758110

[3] Taniguchi T Hamasaki A Okamoto M A case of suspected lymphocytic hypophysitis and organizing pneumonia during maintenance therapy for autoimmune pancreatitis associated with autoimmune thrombocytopenia. *Endocr J* 2006; 53:563–566. 16849834 10.1507/endocrj.k05-179

[4] Wong S Lam WY Wong WK Hypophysitis presented as inflammatory pseudotumor in immunoglobulin G4-related systemic disease. *Hum Pathol* 2007; 38:1720–1723. 17954209 10.1016/j.humpath.2007.06.011

[5] Yang GQ Lu ZH Gu WJ Recurrent autoimmune hypophysitis successfully treated with glucocorticoids plus azathioprine: a report of three cases. *Endocr J* 2011; 58:675–683. 21666339 10.1507/endocrj.k10e-334

[6] Beressi N Beressi JP Cohen R Lymphocytic hypophysitis: a review of 145 cases. *Ann Med Interne (Paris)* 1999; 150:327–341. 10519020

[7] Buxton N Robertson I Lymphocytic and granulocytic hypophysitis: a single centre experience. *Br J Neurosurg* 2001; 15:242–246. 11478060 10.1080/02688690120057664

[8] McKeel DW Common histopathologic and ultrastructural features in granulomatous and lymphoid adenohypophysitis. *Endocrinology* 1983; 112 suppl:190.

[9] Taylon C Duff TA Giant cell granuloma involving the pituitary gland. Case report. *J Neurosurg* 1980; 52:584–587. 7373382 10.3171/jns.1980.52.4.0584

[10] Unlu E Puyan FO Bilgi S Granulomatous hypophysitis: presentation and MRI appearance. *J Clin Neurosci* 2006; 13:1062–1066. 17113990 10.1016/j.jocn.2005.11.046

[11] Nussbaum CE Okawara SH Jacobs LS Lymphocytic hypophysitis with involvement of the cavernous sinus and hypothalamus. *Neurosurgery* 1991; 28:440–444. 2011230 10.1097/00006123-199103000-00019

[12] Stelmachowska M Bolko P Waśko R Lymphocytic hypophysitis and hypothalamitis—case report. *Endokrynol Pol* 2006; 57:648–653. 17253439

[13] Wang XL Lu JM Yang LJ A case of relapsed autoimmune hypothalamitis successfully treated with methylprednisolone and azathioprine. *Neuro Endocrinol Lett* 2008; 29:874–876. 19112420

[14] Kim JE Kim DG Paek SH Stereotactic biopsy for intracranial lesions: reliability and its impact on the planning of treatment. *Acta Neurochir* 2003; 145:547–555. 12910397 10.1007/s00701-003-0048-8

[15] Aker FV Hakan T Karadereler S Accuracy and diagnostic yield of stereotactic biopsy in the diagnosis of brain masses: comparison of results of biopsy and resected surgical specimens. *Neuropathology* 2005; 25:207–213. 16193837 10.1111/j.1440-1789.2005.00634.x

